# Aquaporin-5: A Marker Protein for Proliferation and Migration of Human Breast Cancer Cells

**DOI:** 10.1371/journal.pone.0028492

**Published:** 2011-12-01

**Authors:** Hyun Jun Jung, Ji-Young Park, Hyo-Sung Jeon, Tae-Hwan Kwon

**Affiliations:** 1 Department of Biochemistry and Cell Biology, School of Medicine, Kyungpook National University, Taegu, South Korea; 2 Department of Pathology, School of Medicine, Kyungpook National University, Taegu, South Korea; Ghent University, Belgium

## Abstract

Aquaporin (AQP) is a family of transmembrane proteins for water transport. Recent studies revealed that AQPs are likely to play a role in tumor progression and invasion. We aimed to examine the potential role of AQP5 in the progression of human breast cancer cells. Expression of AQP5 mRNA and protein was seen in human breast cancer cell line (both MCF7 and MDA-MB-231) by RT-PCR and immunoblotting analysis. Immunoperoxidase labeling of AQP5 was observed at ductal epithelial cells of human breast tissues. In benign tumor, AQP5 labeling was mainly seen at the apical domains of ductal epithelial cells. In contrast, in invasive ductal carcinoma, prominent AQP5 labeling was associated with cancer cells, whereas some ducts were unlabeled and apical polarity of AQP5 in ducts was lost. Cell proliferation (BrdU incorporation assay) and migration of MCF7 cells were significantly attenuated by lentivirus-mediated AQP5-shRNA transduction. Hyperosmotic stress induced by sorbitol treatment (100 mM, 24 h) reduced AQP5 expression in MCF7 cells, which was also associated with a significant reduction in cell proliferation and migration. Taken together, prominent AQP5 expression in breast cancer cells with the loss of polarity of ductal epithelial cells was seen during the progression of breast carcinoma. shRNA- or hyperosmotic stress-induced reduction in AQP5 expression of MCF7 cells was associated with significantly reduced cell proliferation and migration. In conclusion, AQP5 overexpression is likely to play a role in cell growth and metastasis of human breast cancer and could be a novel target for anti-breast cancer treatment.

## Introduction

Water channel protein aquaporin (aquaporin: AQP) is a family of transmembrane proteins for water transport, and expression of 13 subtypes has been reported in mammals [1]. AQPs regulate cellular water transport and cell volume and play a key role in body water homeostasis. Some subtypes of AQPs (called as aquaglyceroporins) are also involved in the transport of other molecules such as glycerol and urea [2]. In addition, AQPs mediate signals as membrane proteins by transporting signaling molecules or coupling with other proteins [3], [4]. Importantly, recent studies revealed that AQPs are recognized as the targets for novel anti-tumor therapy, since they are likely to play a role in the promotion of tumor progression and invasion [5], [6], [7].

Altered expression of AQPs has been revealed in several types of tumors upon their specific tissue localization. AQP1, AQP4, and AQP9 are mainly expressed in brain tumor [8], [9], and among them AQP4 is particularly important due to its up-regulation in malignant tumor and brain edema [10]. In the studies of AQP3-null mice, *AQP3* gene deletion induces the resistance to carcinogen-induced skin tumor [6]. Glycerol transport through AQP3 also contributes to the generation of ATP for cell proliferation and tumorigenesis [6]. Moreover, AQP1 is widely over-expressed in tumor tissues of brain, lung, prostate, and colon [7], [11], [12], [13], and AQP3 and AQP5 are also expressed in the colorectal carcinoma [7].

In particular, AQP5 expression in colon cancer cell lines and human colon cancer tissues is associated with cell proliferation and metastasis to liver [14], suggesting that altered expression of AQP5 could play a role in tumor progression [14], [15], [16], [17], [18]. Consistent with this, Ras signal transduction was suggested for enhanced cell proliferation in AQP5-overexpressed in NIH3T3 cells [4]. Moreover, a study for the molecular interaction between AQP5 and its downstream pathway leading to cell invasion revealed that AQP5 binds to SH3 domains of c-Src, a non-receptor cytoplasmic tyrosine kinase associated with invasive and metastatic phenotype in various tumors [15].

Shillingford, *et al.* demonstrated the immunolocalization of AQP5 in the ductal epithelial cells of mouse mammary gland [19]. Since AQP5 is highly expressed in the mammary tumor library, AQP5 may be an important marker protein involved in tumorigenesis and progression. However, the role of AQP5 expression in human breast tissue has not been studied. In this study, we aimed to examine the potential role of AQP5 in the progression of human breast cancer cells by studying 1) the expression of AQP5 in human breast cancer cells (MCF7 and MDA-MB-231 cell lines) and the immunolocalization of AQP5 in human breast cancer tissues; 2) the changes of cellular and subcellular localization of AQP5 in the tissues from benign tumor and invasive ductal carcinoma with or without lymph node (LN) metastasis in human patients; 3) the changes of AQP5 expression related to breast cancer grade; 4) the effects of AQP5 knockdown by lentivirus-mediated shRNA transduction on the cell proliferation and migration of human breast cancer cells (MCF7 cells); and 5) the effects of altered tumor microenvironment (i.e., extracellular hyperosmolality induced by sorbitol treatment) on the AQP5 expression, cell proliferation and migration of human breast cancer cells (MCF7 cells).

## Results

### AQP5 expression in human breast cancer cells

**Figure 1:**
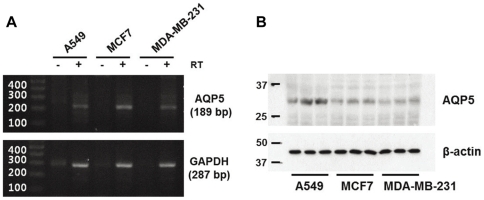
Expression of AQP5 mRNA and protein in human breast cancer cells. **A**) Products of the predicted sizes were generated by RT-PCR of AQP5 (189-bp) and GAPDH (287-bp) in human breast cancer cells (MCF7 and MDA-MB-231), and human lung cancer cells (A549). Negative controls included the omission of reverse transcriptase (- RT). -; Reverse Transcriptase (RT)-negative; +; Reverse Transcriptase (RT)-positive. **B**) Expression of AQP5 protein was investigated in total cell lysate of breast cancer cells. Immunoblotting analysis of AQP5 (32 kDa) and β-actin (43 kDa) was performed in lysates of ERα-positive MCF7 and ERα-negative MDA-MB-231 cells.

Expression of AQP5 mRNA and protein was investigated in human breast cancer cell lines (both MCF7 and MDA-MB-231 cells) by reverse transcriptase-PCR (RT-PCR) and immunoblotting analysis. RT-PCR analysis revealed the AQP5 products (189 bp) in both MCF7 and MDAMB-231 cells (Fig. 1A). The reaction without RT was used as a negative control and human lung cancer cell line A549, the AQP5-expressing cancer cell line [20], was used as a positive control for AQP5 expression (Fig. 1A). Immunoblotting analysis also revealed that both human breast cancer cell lines express AQP5 protein (Fig. 1B).

### Immunohistochemistry of AQP5 in human breast cancer

**Figure 2:**
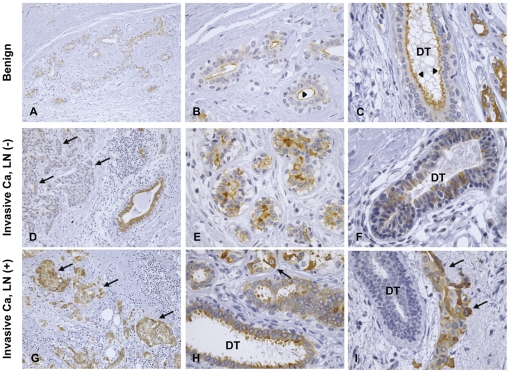
Immunohistochemistry of AQP5. Immunoperoxidase labeling of AQP5 on the sections of tissues from benign breast tumor (A–C), and invasive ductal carcinoma of breast in human patients (D–I; lymph node-negative (D–F) and lymph node-positive (G–I). DT, duct.

In the tissue sections of benign breast tumor, immunoperoxidase labeling of AQP5 was exclusively associated with ductal epithelial cells (Fig. 2A–C). AQP5 labeling in the ductal epithelial cells was mainly in the apical plasma membrane domain (arrowheads in Fig. 2B, C). In contrast, in the tissue sections of invasive ductal carcinoma of breast (Fig. 2D**–**I), AQP5 labeling was also associated with invasive tumor cells (arrows in Fig. 2D, G–I), in addition to the ductal epithelial cells. More prominent AQP5 labeling was associated with invasive cancer cells, particularly in the invasive ductal carcinoma with lymph node (LN) metastasis (arrows in Fig. 2G–I), whereas the labeling was weaker in the invasive ductal carcinoma without lymph node metastasis (arrows in Fig. 2D). The apical polarity of AQP5 labeling in ductal epithelial cells was rarely seen (Fig. 2F, H) and the labeling pattern became diffuse intracellularly (Fig. 2H). Moreover, in the breast cancer tissues with LN metastasis some ducts were unlabeled (Fig. 2I). Thus, the results demonstrated an intense AQP5 labeling in the invasive cancer cells combined with the gradual decrease of AQP5 labeling in the ductal cells, along with the loss of AQP5 polarity in the apical plasma membrane domain during the progression of breast carcinoma.

Importantly, semi-quantitative measurement of AQP5 labeling intensity demonstrated that AQP5 labeling intensity was more prominent in the invasive tumor cells when LNs were involved (Table 1). Moreover, the approximate numbers of AQP5-positive tumor cells among the total tumor cells were increased (Table 1). In contrast, the tumor grade based on the modified Bloom-Richardson-Elston grading system was not different between LN (+) invasive carcinoma and LN (-) invasive carcinoma (Table 1).

**Table 1:** Tumor grade and semi-quantitative measurement of AQP5 labeling intensity and proportion in the invasive ductal carcinoma with or without lymph node metastasis.

Type	Patients	Tumor grade*	Tubule formation /Nuclear grade /Mitotic rate	AQP5 labeling intensity	Proportion of AQP5 labeling
Lymph node-	1	3	3/3/3	-	-
negative invasive	2	2	3/2/2	-	-
Carcinoma	3	3	3/2/3	2	3
	4	2	3/2/1	-	-
	5	1	3/1/1	3	3
	6	3	3/3/3	2	2
	7	1	2/2/1	-	-
	8	2	3/2/1	-	-
	9	2	3/2/2	N.A.	N.A.
	10	2	3/2/1	N.A.	N.A.
Lymph node-	1	2	3/2/2	1	2
positive invasive	2	3	3/3/2	2	2
Carcinoma	3	2	3/2/1	2	3
	4	3	3/3/3	2	2
	5	2	3/2/1	3	3
	6	2	3/2/2	3	3
	7	1	1/2/1	1	1
	8	2	3/2/2	3	3
	9	2	3/3/1	3	3
	10	2	3/2/2	3	3

Tumor grade was determined by modified Bloom-Richardson-Elston grading system. Immunolabeling intensity of AQP5 was scored as negative (-), weak (score 1), moderate (score 2), and strong (score 3) by expert pathologists. Proportion was the approximate numbers of AQP5-positive tumor cells among the total tumor cells, scored as negative(-), <33% (score 1), 34%–66% (score 2), and>67% (score 3). N.A., not available.

### Inhibition of cell proliferation and migration of MCF7 cells in response to shRNA-mediated knockdown of AQP5

**Figure 3:**
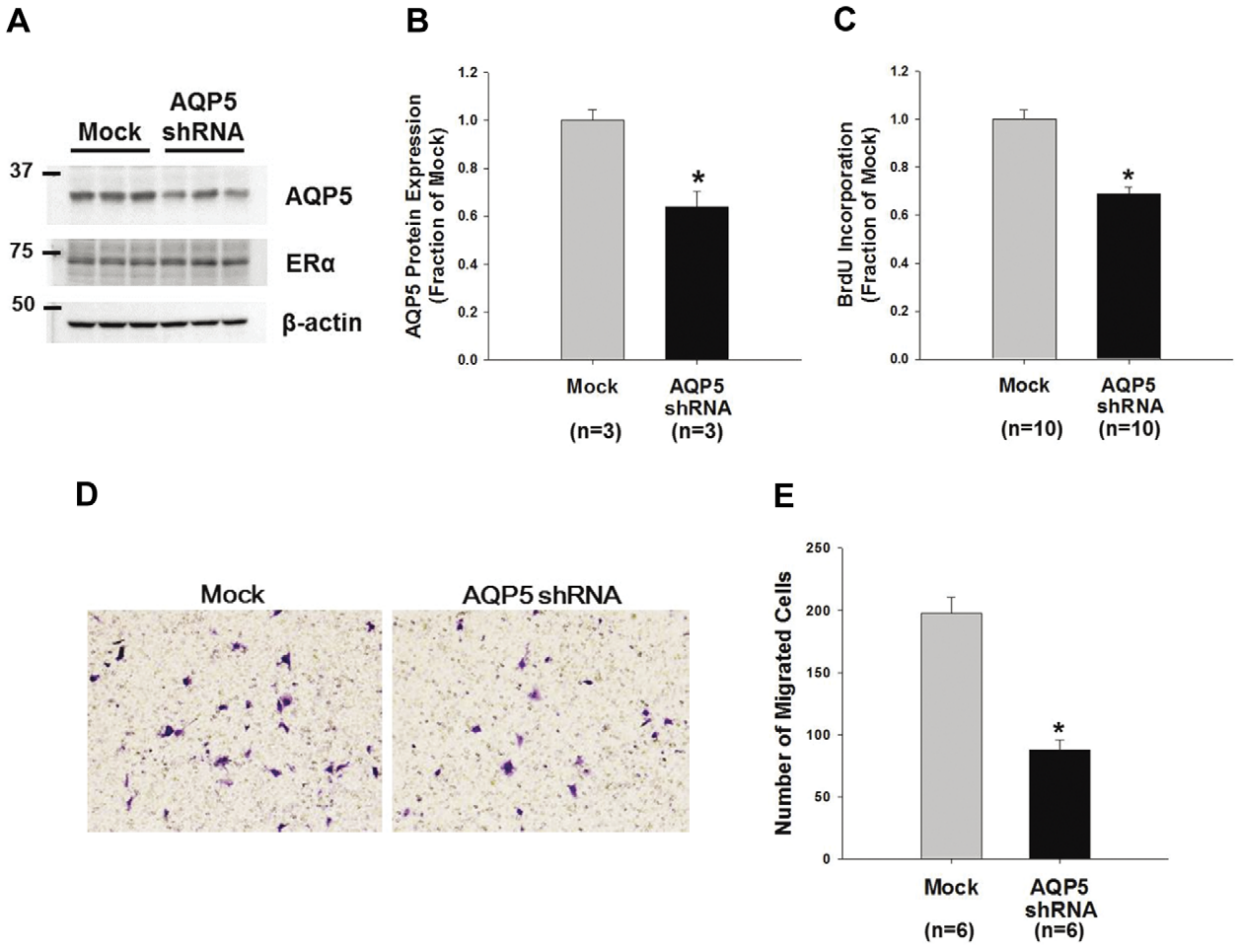
Changes in proliferation and migration of MCF7 cells infected by lentivirus containing human AQP5 shRNA. **A and B**) Semi-quatitative immunoblotting of AQP5. AQP5 expression was significantly decreased in the AQP5 shRNA-infected MCF7 cells, compared to the mock shRNA-infected MCF7 cells. n, number of MCF7 cell lysates. **C**) BrdU cell proliferation assay. AQP5 shRNA- or mock shRNA-infected MCF7 cells were incorporated with BrdU for 24 h, and ELISA demonstrated a significant decrease of BrdU incorporation in the MCF7 cells with AQP5 knockdown. **D**) Cell migration assay for MCF7 cells with AQP5 knockdown. MCF7 cells with AQP5 knockdown were incubated in upper chamber of 24-transwell plate for 4 h, and migration was induced by FBS-gradient medium. Migrated cells were stained by crystal violet solution and counted in five different fields (×100) per well. Value of migrated cells was described as the total cell number in five random fields, and two-independent experiment was performed. *P<0.05 when compared to mock shRNA-infected control group.

To examine whether AQP5 expression plays a role in the progression of breast cancer in human patients, changes of proliferation and migration of breast cancer cells were examined in response to shRNA-mediated knockdown of AQP5. AQP5 protein expression in MCF7 cells was significantly decreased by lentivirus-mediated AQP5 shRNA transduction (64±7% of control level, *P*<0.05, Fig. 3A and B), whereas ERα expression was unchanged. BrdU cell proliferation assay demonstrated that BrdU incorporation was significantly decreased (69±2% of mock control, *P*<0.05, Fig. 3C) in MCF7 cells with AQP5 knockdown, indicating that AQP5 knockdown in human breast cancer cells was associated with decreased cell proliferation. Moreover, migrating ability of MCF7 cells was examined by FBS gradient-induced cell migration assay. The number of migrated MCF7 cells was significantly decreased (47±4% of mock control, *P*<0.05, Fig. 3D, E) in response to AQP5 knockdown.

### Inhibition of AQP5 expression in MCF7 cells in response to sorbitol treatment

**Figure 4:**
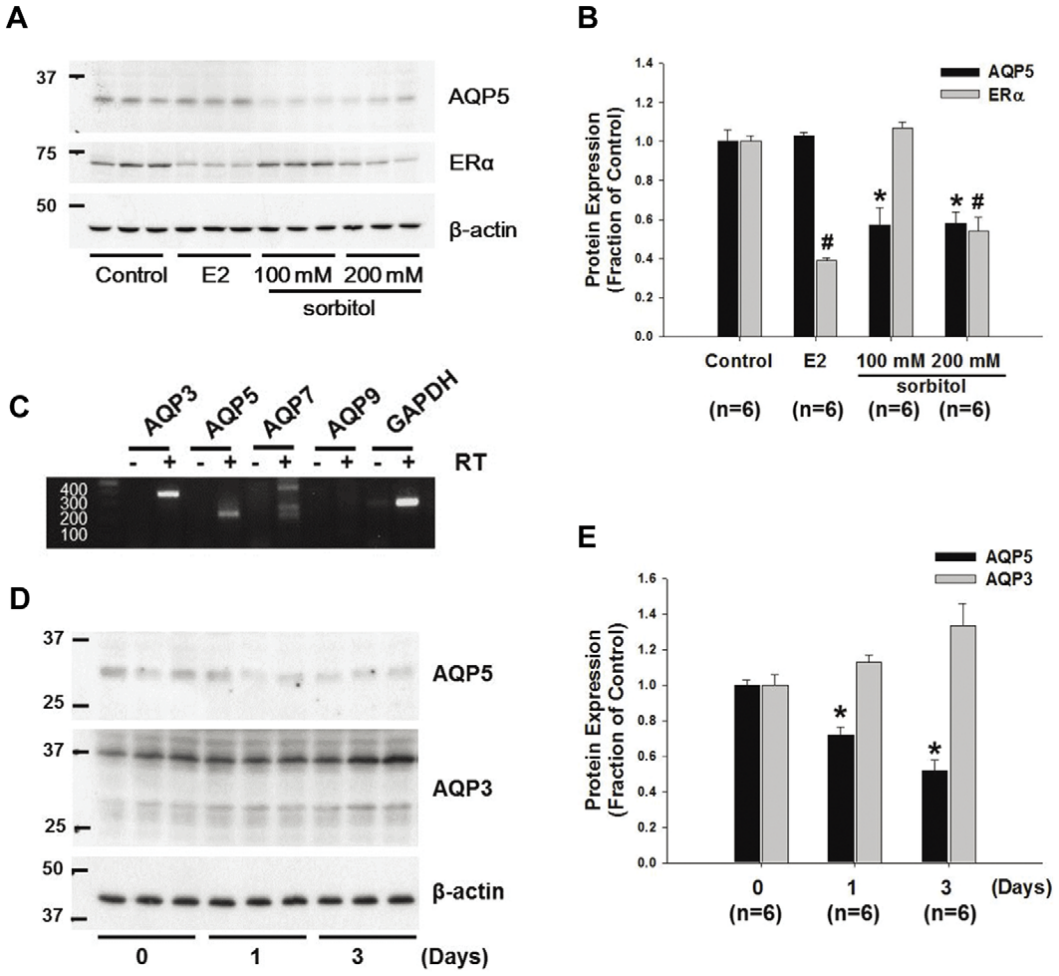
Changes in expression of AQP5, ERα, and AQP3 in MCF7 cells by sorbitol-induced hyperosmotic stress. **A and B**) Semiquantitative immunoblotting of AQP5 and ERα in MCF7 cells treated by vehicle, E2 (10^−9^ M), and sorbitol (100 mM or 200 mM) for 24 h. n, number of MCF7 cell lysates. **C**) RT-PCR of AQP3, -5, -7, -9, and GAPDH in human breast cancer cell line, MCF7. The primer sequences are described in Table S2. Negative controls included the omission of reverse transcriptase (- RT). -; Reverse Transcriptase (RT)-negative; +; Reverse Transcriptase (RT)-positive. **D and E**) Semiquantitative immunoblotting of AQP3 and AQP5 in MCF7 cells treated by 100 mM sorbitol for 1 d or 3 d. *P<0.05 when compared to vehicle-treated control group (AQP5), and ^#^ *P*<0.05 when compare to vehicle-treated control group (ERα).

Hyperosmotic stress is known to change AQPs expression in various cells [21], [22], [23]. The change of AQP5 expression in breast cancer cells was examined in response to extracellular hyperosmotic stress induced by sorbitol-containing medium. MCF7 cells were incubated with either 100 mM or 200 mM sorbitol for 24 h, which osmolality was 380 or 460 mOsm/KgH_2_O, respectively. Expression of AQP5 protein was significantly decreased in the cells exposed to either 100 mM (57±9%, *P*<0.05, Fig. 4A and B) or 200 mM sorbitol medium (58±6%, *P*<0.05, Fig. 4A and B), compared with the cells maintained under isosmotic condition (300 mOsm/KgH_2_O). To examine whether culture medium containing sorbitol induced non-specific downregulation of proteins, change of ERα expression was also examined (Fig. 4). ERα expression in the MCF7 cells was not affected in response to 100 mM sorbitol medium, but decreased in 200 mM sorbitol medium, indicating that hyperosmotic stress induced by 100 mM sorbitol for 24 h caused a selective decrease of AQP5 expression in MCF7 cells. In addition, 17β-estradiol (E2) treatment (10^−9^ M, 24 h) in MCF7 cells induced a significant decrease of ERα expression in MCF7 cells, as previously demonstrated [24].

Moreover, RT-PCR analysis revealed that AQP3 and AQP7 mRNA expression was also observed in human breast cancer MCF7 cells, in addition to AQP5 expression (Fig. 4C). Since it was reported that AQP3 expression in cultured human keratinocytes was increased under hypertonic condition [21], the changes of protein expression of AQP3 and AQP5 were studied in MCF7 cells under hyperosmotic environment (380 mOsm/KgH_2_O) for 1 day or 3 days induced by 100 mM sorbitol. As shown in Figure 4 (panel D and E), hyperosmotic stress by sorbitol significantly reduced AQP5 expression in time-dependent manner, whereas AQP3 expression was unchanged, revealing that sorbitol-induced hyperosmotic stress induced a selective decrease of AQP5 expression in MCF7 cells.

### Inhibition of proliferation and migration of MCF7 cells in response to sorbitol-induced hyperosmotic stress

**Figure 5:**
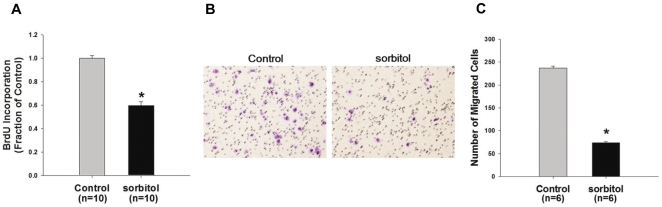
Change of proliferation and migration of sorbitol-treated MCF7 cells. **A**) BrdU cell proliferation assay. MCF7 cells incubated in 100 mM sorbitol medium for 24 h were incorporated with BrdU for another 24 h. **B and C**) Cell migration assay for sorbitol-treated MCF7 cells. *P<0.05 when compared to vehicle-treated control group.

To test the effects of sorbitol-induced hyperosmotic stress and hence the selective decrease of AQP5 expression on the cell proliferation, MCF7 cells were incorporated by BrdU for another 24 h in the presence of 100 mM sorbitol after an incubation with 100 mM sorbitol for 24 h. Sorbitol-treated MCF7 cells showed significantly reduced BrdU incorporation (60±3% of control levels, *P*<0.05, Fig. 5A). Moreover, migration of MCF7 cells was also inhibited by 100 mM sorbitol treatment (31±3% of control levels, *P*<0.05, Fig. 5B–C). Therefore, hyperosmotic stress by 100 mM sorbitol and hence selective AQP5 downregulation could decrease the cell proliferation and migration of human breast cancer cells (ERα-positive MCF7 cells).

## Discussion

In this study, we demonstrated that AQP5 is likely to play a role in proliferation and migration of human breast cancer cells. RT-PCR and immunoblotting analysis revealed the expression of AQP5 mRNA and protein in human breast cancer cells and immunohistochemistry demonstrated AQP5 labeling in the ductal epithelial cells of human breast tissues. Importantly, prominent AQP5 labeling was associated with invasive cancer cells combined with the gradual decrease of AQP5 labeling intensity in the ductal epithelial cells during the progression of breast carcinoma. Moreover, AQP5 polarity in the apical plasma membrane domain of the ductal epithelial cells was lost. Semi-quantitative assessment of AQP5 labeling intensity in the invasive cancer cells demonstrated that AQP5 labeling intensity was significantly higher in the invasive carcinoma with LN metastasis, compared with the one without LN metastasis, despite no differences in tumor grade between the two groups. Cell culture experiments also revealed the role of AQP5 in tumor cell proliferation and migration. Lentivirus-mediated AQP5-shRNA transduction or sorbitol-induced hyperosmotic stress in human breast cancer cells resulted in the significant reduction of AQP5 expression, which was associated with markedly decreased cell proliferation and migration.

Recent studies suggest that AQP is regarded as an important oncogenic factor for tumor progression [5], [6], [7], [10]. It has been reported that AQP5 is expressed in the ductal epithelial cells of various secretory tissues, such as lung and salivary glands [15], [25], [26]. In the present study, AQP5 mRNA and protein are expressed in human breast cancer cell lines, MCF7 and MDA-MB-231, both of which are originated from breast ductal epithelial cells. Moreover, immunohistochemistry revealed the localization of AQP5 at the ductal epithelial cells of human breast cancer tissues. Since tumorigenesis occurs in ductal epithelial cells, and some begin in lobular cells of breast [27], [28], the observed localization of AQP5 suggests that it may play an important role in tumorigenesis and progression of ductal carcinoma of breast.

Importantly, we demonstrated that intense AQP5 labeling was seen in the invasive ductal carcinoma cells in human patients exhibiting LN metastasis. This finding is compatible with several previous studies revealing that AQP5-overexpressing cancer cells exhibit increased ability of cell proliferation and migration, e.g., chronic myelogenous leukemia [16], colorectal carcinoma [14], and non small cell lung carcinoma (NSCLC) [15]. Thus, increased AQP5 expression in the invasive ductal carcinoma of breast could suggest its role in the promotion and cell growth of cancer cells.

Consistent with this, previous studies demonstrated that AQP5-overexpressing cancer cells exhibit the increased ability of cell proliferation and migration *via* several different signaling pathways. Zhang, *et al.* [29] demonstrated that epidermal growth factor receptor, extracellular receptor kinases, and p38 mitogen-activated protein kinase pathways were activated in lung cancer cell line SPC-A1 and PC-9 stably transfected with AQP5, resulting in the promotion of cell proliferation and migration. They also showed significantly increased PCNA and c-myc expression in AQP5 transfected cells (28). In addition, MUC5AC mucin expression was increased, which might be involved in lung cancer metastasis (28). AQP5-overexpressed NIH3T3 cells also exhibited cell proliferation *via* Ras signal transduction (4), and invasive and metastatic phenotype in various tumors was associated with AQP5 binding to SH3 domains of c-Src, a non-receptor cytoplasmic tyrosine kinase (15).

In contrast, shRNA-mediated AQP5 knockdown in SPC-A1 cells was associated with decreased cell migration and invasion, presumably by changing cell shape and volume through the decreased osmotic water permeability of cell membrane [30]. In the present study, we also demonstrated significantly decreased cell proliferation and migration of human breast cancer cells with shRNA- or hyperosmotic stress-mediated AQP5 knockdown. Osmolar changes have been revealed to affect to cell proliferation, as previously demonstrated by the negative effect of hypertonicity on cell proliferation[31]. Kato, *et al.* [32] also demonstrated that alkaline phosphatase activity was increased in MCF7 cells by hyperosmolar stress, and the increased alkaline phosphatase activity was known to be associated with reduction of cell proliferation [33], [34]. In the present study, sorbitol treatment suppressed the migration and proliferation of breast cancer cells, where AQP5 expression was significantly decreased, but not AQP3. The results suggested that tumor microenvironment, particularly osmolar condition, could be importantly involved in the proliferation and migration of breast cancer cells. Further studies are required to elucidate the specific underlying molecular mechanisms for 1) increased AQP5 expression with loss of subcellular polarity of expression in the invasive carcinoma; and 2) the direct association between up-regulated AQP5 expression and progression of cancer cells.

In summary, prominent AQP5 expression was seen in the cancer cells with the loss of polarity of ductal epithelia during the progression of invasive ductal carcinoma of breast. More prominent AQP5 labeling intensity of the cancer cells was seen in the invasive carcinoma with LN metastasis than that of the invasive carcinoma without LN metastasis, whereas there was no difference in the tumor grade between the two groups. shRNA- or hyperosmotic stress-induced reduction of AQP5 expression was associated with significantly reduced cell proliferation and migration, demonstrated by *in vitro* cell culture experiments of MCF7 cells. Therefore, AQP5 expression could be a novel biomarker for cell growth and metastasis of human breast cancer, and hence AQP5 might be a critical target for anti-breast cancer treatment.

## Materials and Methods

### Cell line and Tissue specimen from human patients

Breast cancer cell lines MCF7 (HTB-22) and MDA-MB-231 (HTB-26) were obtained from the American Type Culture Collection (Manassas, VA), and cells were cultured in DMEM medium containing 0.1% penicillin-streptomycin solution and 10% heat-inactivated FBS at 37°C.

Immunohistochemical examination of AQP5 on the tissue sections were performed using paraformaldehyde-immersion fixed and paraffin-embedded tissues of benign breast tumor and invasive ductal carcinoma (Table 2), obtained from Department of Pathology, Kyungpook National University Hospital. The breast tumor samples were obtained from 2000 to 2010. Theses tissues included 10 benign tumors and 20 invasive ductal carcinoma (10 non-LN metastases and 10 LN metastases, Table 2). Tumor grade was determined by modified Bloom-Richardson-Elston grading system.

**Table 2:** Clinical characteristics of breast cancer patients from three different groups.

Category	Benign tumor	Lymph node negativeInvasive ductal carcinoma	Lymph node positiveInvasive ductal carcinoma
Age	18–55 (mean: 43.6)	36–66 (mean: 50.3)	39–64 (mean: 47.7)
Tumor size (cm)	-	>10 mm but≤20 mm in greatest dimension (T1c) (n = 5)>20 mm but≤50 mm in greatest dimension (T2) (n = 5)	>10 mm but≤20 mm in greatest dimension (T1c) (n = 2)>20 mm but≤50 mm in greatest dimension (T2) (n = 8)
Stage	-	IA (n = 5)IIA (n = 5)	IIA (n = 1)IIB (n = 7)IIIC (n = 2)
Diagnosis	Fibrocystic change (n = 6)Fibroadenoma (n = 2)Intraductal papilloma (n = 1)Gynecomastia (n = 1)	Invasive ductal carcinoma (n = 9)Invasive pleomorphic lobular carcinoma (n = 1)	Invasive ductal carcinoma (n = 10)

n, number of patients in each group.

### Ethics statement

Paraformaldehyde-immersion fixed and paraffin-embedded tissues of breast tumor were obtained from Department of Pathology, Kyungpook National University Hospital under the approval of the Institutional Review Board (IRB No. 2011-04-015). The H&E stained slides were re-evaluated anonymously by pathologists. Informed consent from all patients involved in this study was not obtained, since the data were evaluated anonymously. The ethics committee of IRB, Kyungpook National University Hospital, specifically waived the need for consent.

### RT-PCR analysis of AQP5

Total RNA from human breast cancer cell lines was extracted using TRI reagent (Molecular Research Center, Cincinnati, OH). Total RNA was used and one-step RT-PCR was performed using Access RT-PCR system (Promega, Medison, WI) according to the manufacturer's instructions [35]. For RT-PCR, the following components were added to the reaction vials: 780 ng of total RNA, both sense and antisense primers, AMV/Tfl 5×reaction buffer, dNTP mix, 25 mM MgSO_4_, AMV reverse transcriptase, Tfl DNA polymerase, and DEPC-treated water in a total volume of 50 µl. The samples were incubated for 45 min at 45°C, thereafter RT was terminated by heating at 94°C for 2 min. And then, the samples were subjected to thermal cycling for the second strand cDNA synthesis and amplification. Amplification was performed for 40 cycles with 30 s/94°C denaturation, 1 min/60°C annealing, and 2 min/68°C extension. An amplification of the mRNA for housekeeping gene hGAPDH was used as an internal control. The primer sequences were as follows: hAQP5 sense; 5′- CAGCTGGCACTCTGCATCTT-3′, antisense; 5′-TGAACCGATTCATGACCACC-3′, hGAPDH sense; 5′-GCCAAAAGGGTCATCATCTC-3′, antisense; 5′-GTAGAGGCAGGGATGATGTTC-3′. RT-PCR products were electrophoresed on 1.5% agarose gel containing ethidium bromide.

### AQP5 knockdown using lentivirus containing AQP5 short hairpin RNA

Short hairpin RNA (shRNA) set (RHS4533-NM_001651) for human AQP5 (accession: NM_001651) were purchased from Open biosystems (Huntsville, AL). The sequences for AQP5 shRNA are listed in Table S1. Lentivirus containing AQP5 shRNA was prepared using the lentiviral packaging system (ViraPower Lentiviral Expression System, Invitrogen), as described previously [36]. Viral DNA and AQP5 shRNA-containing pLKO.1 vector were transfected into 293FT cells and incubated for 4 days. Then, the supernatant of 293FT cell cultures containing viral particles (1/5 volume of fresh breast cancer cell culture medium) was added to breast cancer cells cultured in 3 different well plates (n = 3 in Fig 3A) with 16 ng/mL polybrene. After incubation for 2 days, cells were screened in the medium containing puromycin (2 µg/mL) for 2 weeks.

During this screening procedure, the proliferation of MDA-MB-231 cells were markedly retarded compared to MCF7 cells and hence MDA-MB-231 cells were not collected for doing further experiments. After selection of MCF7 cells, protein expression of AQP5 was analyzed by immunoblotting. Lentiviral pLKO.1 vector transduced-cells were the negative control (Mock). The number of replicates in the figures (Fig. 3, 4, 5) indicates the number of cell lysate preparations from independent experiments.

### Immunoblotting analysis

The cell lysates were obtained in RIPA buffer (10 mM Tris-HCl, 0.15 M NaCl, 1% NP-40, 1% Na-deoxycholate, 0.5% SDS, 0.02% sodium azide, 1 mM EDTA, pH 7.4) including proteinase and phosphatase inhibitors (0.4 µg/ml leupeptin, 0.1 mg/ml pefabloc, 1 mM Na_3_VO_4_, 25 mM NaF, and 0.1 µM okadaic acid). Immunoblotting was performed as previously described [37]. The total protein concentration was measured (Pierce BCA protein assay reagent kit; Pierce, Rockford, IL) and all samples were adjusted with RIPA buffer to reach the identical final protein concentrations and solubilized at 65°C for 15 minutes in Laemmli sample buffer, and then stored at 4°C. Primary antibodies used were anti-AQP5 (1:1,000, Abcam), anti-estrogen receptor α (1:1,000, Cell Signaling) and anti-β-actin (1:100,000, Sigma). The film was scanned (EPSON Perfection V700 Scanner, Long Beach, CA) and band density was quantitated by Image J (NIH Image, NIMH, NIH, Bethesda, MD). The densitometry values for each protein were corrected by densitometry of β-actin and were normalized to facilitate comparisons.

### Immunohistochemistry

Paraffin-embedded blocks of 4% paraformaldehyde (PFA)-fixed human breast tumor tissues (IRB No. 2011-04-015, Kyungpook National University Hospital) were prepared for 2 µm thickness sections on the slides. The sections were labeled by anti-AQP5 antibody (1∶200, Abcam) at 4°C overnight, then by a horseradish peroxidase (HRP)-conjugated goat anti-rabbit secondary antibody (1:200, P448; DAKO) for 90 min at room temperature, as previously described [26]. AQP5 immunolabeling was reviewed by expert pathologists and the intensity and proportion were scored. Immunolabeling intensity of AQP5 was scored as negative (-), weak (score 1), moderate (score 2), and strong (score 3). Proportion is the approximate numbers of AQP5-positive tumor cells among the total tumor cells, scored as negative (-),<33% (score 1), 34%–66% (score 2), and >67% (score 3).

### BrdU cell proliferation assay

BrdU cell proliferation assay was carried out according to the manufacturer's instructions (QIA58, Calbiochem). Briefly breast cancer cells (1×10^4 ^MCF7 cells/well), either lentiviral pLKO.1 vector transduced-cells (Mock, n = 10 in Fig 3C) or lentivirus containing AQP5 shRNA-infected cells (AQP5 shRNA, n = 10 in Fig 3C), were allowed to attach to the culture plate at 37°C overnight. For BrdU incorporation, culture media was changed to fresh culture media containing BrdU (1:2,000) and incubated for 24 h at 37°C. After washing, fixative/denaturing solution was added to each well and kept for 30 min at room temperature. The cells were then treated by anti-BrdU antibody (1:100) for 1 h at room temperature. For conjugation of peroxidase goat anti-mouse IgG to anti-BrdU antibody, 100 µL of secondary antibody conjugate solution was added into each well. Free conjugates were removed by washing with wash buffer three times and distilled water. Then, after removal of contents of wells, substrate solution was added to each well and incubated for 15 min in the dark at room temperature. The reaction between HRP and substrates was stopped by addition of stop solution. Absorbance of each well was measured using a spectrophotometric plate reader at wavelength of 520 nm. BrdU incorporation was calculated by the following equation: BrdU incorporation = [(absorbance of treated well) - (absorbance of non-BrdU labeled well)]/[(absorbance of control well) - (absorbance of non-BrdU labeled well)].

### Cell migration assay

MCF7 cells were studied in a permeable filter of transwell system (8 µm pore size, Transwell® Permeable Supports, Cat. No. 3422, Corning Incorporated, MA). For coating the membrane of inserts, the side of membrane facing lower chamber of the inserts was coated with 10 ng/mL collagen IV solution (C5533, Sigma) at 4°C overnight, and blocked by 2% BSA/PBS solution for 1 h at room temperature. After trypsinization, cell suspension (1×10^4^ cells/well) was seeded into the membrane facing upper chamber containing culture medium with 1% FBS. Cell migration to the other side of membrane was induced by 10% FBS-containing medium in the lower chamber for 4 h. Migrated cells were fixed in 4% PFA solution for 10 min and then stained in 0.03% crystal violet solution for 10 min. After removal of un-migrated cells by swab, the stained cells were subjected to microscopic examination. Migrated cells were counted in the five randomly selected fields (×100) per well, respectively.

### Statistical analysis

Values were presented as means ± standard errors. Statistical comparisons were accomplished by unpaired *t* test (when the number of groups were 2) or by one-way analysis of variance (ANOVA) followed by Bonferroni's multiple comparisons test (when the number of groups was more than 2). Multiple comparisons tests were only applied when a significant difference was determined in the ANOVA, *P*<0.05.

## Supporting Information

Table S1 **Sequences of primers for RT-PCR analysis.**
(DOC)

Table S2 **shRNA sequences for** ***AQP5.***
(DOC)
