# Supplementary material for: Aquaporin-5: A Marker Protein for Proliferation and Migration of Human Breast Cancer Cells
Source: PLoS One. 2011 Dec 1;6(12):e28492. doi: 10.1371/journal.pone.0028492 (PMC3228775; doi:10.1371/journal.pone.0028492)
Supplement: Table S1 — Sequences of primers for RT-PCR analysis. (DOC) [file pone.0028492.s001.doc]

**Supporting Information**

**Table S1. Sequences of primers for RT-PCR analysis**

| **Name (Accession)** | **Predicted**  **Product size (bp)** | **Sequences (5’→3’)** |
| --- | --- | --- |
| AQP3 (NM004925.3) | 389 | (sense) TCA ATG GCT TCT TTG ACC AGT TCA  (antisense) CTT CAC ATG GGC CAG CTT CAC ATT |
| AQP5 (NM001651.1) | 189 | (sense) CAG CTG GCA CTC TGC ATC TT  (antisense) TGA ACC GAT TCA TGA CCA CC |
| AQP7 (NM001170.1) | 213 | (sense) GCT GTA ACT GAG AAG CCC CC  (antisense) GAA CTC GGC CAG GAA CTC TC |
| AQP9 (NM020980.3) | 136 | (sense) GTC CTC AGA GAA GCC CCA AG  (antisense) ATC AAG ATG AAC GTG CCC AA |
| GAPDH (NM002046.3) | 287 | (sense) GCC AAA AGG GTC ATC ATC TC  (antisense) GTA GAG GCA GGG ATG ATG TTC |
