# Supplementary material for: Aquaporin-5: A Marker Protein for Proliferation and Migration of Human Breast Cancer Cells
Source: PLoS One. 2011 Dec 1;6(12):e28492. doi: 10.1371/journal.pone.0028492 (PMC3228775; doi:10.1371/journal.pone.0028492)
Supplement: Table S2 — shRNA sequences for AQP5. (DOC) [file pone.0028492.s002.doc]

**Supporting Information**

**Table S2. shRNA sequences for *AQP5***

| **shRNA** | **5’→3’ Sequences** |
| --- | --- |
| shRNA #1 | CCGGCCACCTTGTCGGAATCTACTTCTCGAGAAGTAGATTCCGACAAGGTGGTTTTTG (TRCN0000059633) |
| shRNA #2 | CCGGACGCGCTCAACAACAACACAACTCGAGTTGTGTTGTTGTTGAGCGCGTTTTTTG (TRCN0000059634) |
| shRNA #3 | CCGGTGCGGTGGTCATGAATCGGTTCTCGAGAACCGATTCATGACCACCGCATTTTTG (TRCN0000059636) |
| shRNA #4 | CCGGGCTCCGGGCTTTCTTCTACGTCTCGAGACGTAGAAGAAAGCCCGGAGCTTTTTG (TRCN0000059637) |
